# ADULTS’ UNDERSTANDING OF COGNITIVE DECLINE, DEMENTIA, AND ALZHEIMER’S DISEASE

**DOI:** 10.1093/geroni/igad104.0425

**Published:** 2023-12-21

**Authors:** Laura Mehegan, Chuck Rainville

**Affiliations:** AARP, Washington, District of Columbia, United States; AARP, Washington, District of Columbia, United States

## Abstract

A 2021 nationally representative survey fielded by AARP of adults aged 40 and older (N=3,022) showed that nearly six in ten (58%) believe that cognitive decline is inevitable, and two-thirds (66%) believe they will experience cognitive decline as they age. Nearly half (48%) think it is likely they will get dementia. As a follow up to this research, in 2022, AARP sponsored focus groups (N=31) and in-depth interviews (N=24) to further explore the perceptions of dementia and cognitive decline among individuals aged 40 and older. The main purpose of this research was to evaluate how Americans understand and communicate about dementia and cognitive decline. The results of this research showed personal experience played a greater role than age when it came to participants’ concern and understanding about cognitive decline. Worry about cognitive decline increased for those caring for a friend or family members with dementia or Alzheimer’s disease. Participants expressed feelings of inevitability of cognitive decline, but not dementia or Alzheimer’s disease. Forgetfulness was mentioned by many participants as part of the aging process, but they were clear that dementia and Alzheimer’s disease were not inevitable and were not the same as age-related memory lapses. Normal, age-related changes in memory and cognitive function were not well-understood and neither was the distinction between dementia and Alzheimer’s disease. The results from these studies suggest the general population needs information on cognitive decline, dementia, and Alzheimer’s disease to better understand each and to raise awareness on the proactive steps needed for brain health

